# 2118. Patient Characteristics and Clinical Outcomes of *Leptotrichia* spp. Bacteremia

**DOI:** 10.1093/ofid/ofac492.1739

**Published:** 2022-12-15

**Authors:** Nischal Ranganath, Douglas Challener, Audrey Schuetz, Ryan W Stevens, Joshua D Shirley, Aditya Shah

**Affiliations:** Mayo Clinic College of Medicine and Science, Rochester, Minnesota; Mayo Clinic, Rochester, Minnesota; Mayo Clinic, Rochester, Minnesota; Mayo Clinic, Rochester, Minnesota; Mayo Clinic, Rochester, Minnesota; Mayo Clinic, Rochester, Minnesota

## Abstract

**Background:**

*Leptotrichia* spp. are anaerobic, gram-negative bacilli that are part of the normal oral and intestinal microbiota. Although traditionally considered non-pathogenic, these bacteria can result in invasive infections including bacteremia in immunosuppressed patients, particularly those with neutropenia. There are limited published data to inform best management strategies in those with *Leptotrichia* bacteremia.

**Methods:**

All cases of *Leptotrichia* spp. bacteremia between January 2012 and 2022 at our tertiary academic medical center were retrospectively reviewed to determine patient risk factors, clinical outcomes, and antimicrobial susceptibilities. Descriptive statistical methods were used. Antimicrobial susceptibilities were performed by agar dilution.

**Results:**

26 cases of *Leptotrichia* spp. bacteremia were identified (Figure 1). The mean patient age was 55 years (SD 17), with 9 female patients (35%). All 26 patients were immunocompromised, predominantly due to hematologic malignancy (69%) or hematopoietic stem cell transplant (23%) (HSCT). 25 of 26 patients were actively neutropenic, with a median duration of neutropenia of 21 days (13-26) (Table 1). The most frequent sources of *Leptotrichia* bacteremia were gastrointestinal translocation (60%), followed by catheter-related infection (35%). 10 patients had polymicrobial bacteremia (38.5%). The primary antibiotics utilized to treat *Leptotrichia* bacteremia included metronidazole (42%), piperacillin-tazobactam (27%), and carbapenems (19%). Overall, the mean duration of treatment was 11 days, with a 60-day all-cause mortality of 19% (Table 1) with no cases of microbiologic relapse. In the 22 clinical isolates evaluated for susceptibility, *Leptotrichia* spp. were largely susceptible to metronidazole, penicillin, ertapenem, and piperacillin-tazobactam, but uniformly resistant to moxifloxacin (Table 2).

**Figure 1:**
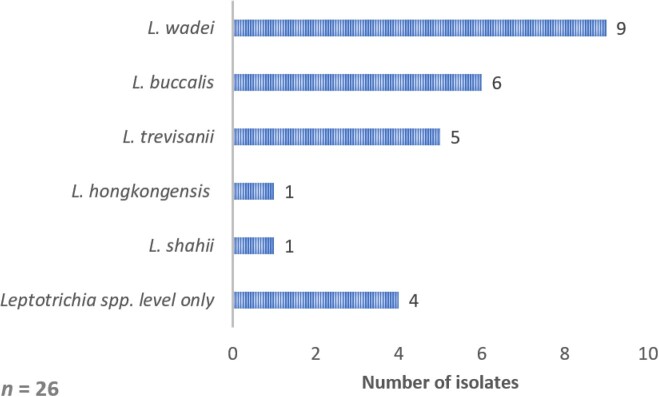
Leptotrichia spp. identified as cause of bacteremia in 26 patients

**Table 1:**
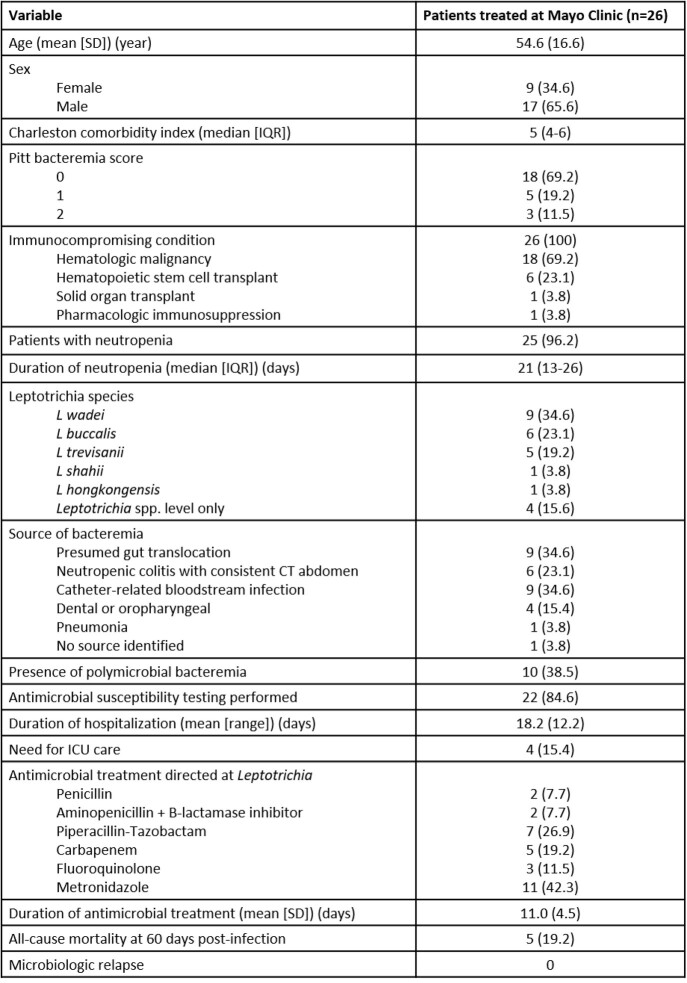
Baseline characteristics of patients with Leptotrichia bacteremia (n=26) between January 2012 to January 2022

**Table 2:**
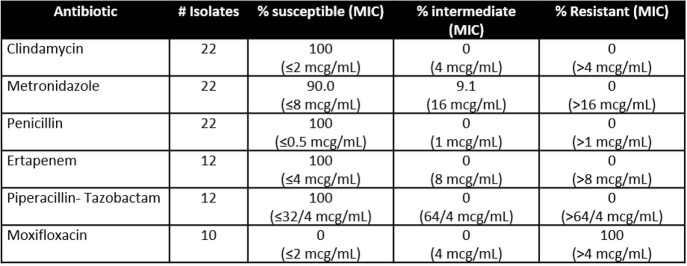
Antimicrobial susceptibility profile of the Leptotrichia spp. (n=22)

**Conclusion:**

*Leptotrichia* spp. may be a rare cause of bacteremia in neutropenic hosts, particularly those with underlying hematologic malignancies and HSCT. The pathogen has a favorable susceptibility profile to penicillins and carbapenems, but has high degree of resistance to fluoroquinolones.

**Disclosures:**

**All Authors**: No reported disclosures.

